# mbQTL: an R/Bioconductor package for microbial quantitative trait loci (QTL) estimation

**DOI:** 10.1093/bioinformatics/btad565

**Published:** 2023-09-14

**Authors:** Mercedeh Movassagh, Steven J Schiff, Joseph N Paulson

**Affiliations:** Department of Biostatistics, Harvard T. H. Chan School of Public Health, Boston, MA 02115, United States; Department of Neurosurgery, Yale University School of Medicine, New Haven, CT 06510, United States; Department of Neurosurgery, Yale University School of Medicine, New Haven, CT 06510, United States; Department of Data Sciences, N-Power Medicine, Redwood City, CA 94063, United States

## Abstract

**Motivation:**

In recent years, significant strides have been made in the field of genomics, with the commencement of large-scale studies aimed at collecting host mutational profiles and microbiome data. The amalgamation of host gene mutational profiles in both healthy and diseased subjects with microbial abundance data holds immense promise in providing insights into several crucial research questions, including the development and progression of diseases, as well as individual responses to therapeutic interventions. With the advent of sequencing methods such as 16s ribosomal RNA (rRNA) sequencing and whole genome sequencing, there is increasing evidence of interplay of human genetics and microbial communities. Quantitative trait loci associated with microbial abundance (mbQTLs), are genetic variants that influence the abundance of microbial populations within the host.

**Results:**

Here, we introduce mbQTL, the first R package integrating 16S ribosomal RNA (rRNA) sequencing and single-nucleotide variation (SNV) and single-nucleotide polymorphism (SNP) data. We describe various statistical methods implemented for the identification of microbe–SNV pairs, relevant statistical measures, and plot functionality for interpretation.

**Availability and implementation:**

mbQTL is available on bioconductor at https://bioconductor.org/packages/mbQTL/.

## 1 Introduction

With the advent of sequencing methods such as 16s ribosomal RNA (rRNA) sequencing and whole genome sequencing, there is increasing evidence of the interplay between human genetics and microbial communities (Qin *et al.* 2022). Quantitative trait loci associated with microbial abundance (mbQTLs), are genetic variants that influence the abundance of microbial populations within the host. mbQTLs are identified through microbiome genome-wide association studies (mGWAS) that compare the genetic profiles of a host to the abundance of bacteria within a given body site (Weissbrod *et al.* 2018). By identifying host single-nucleotide variants (SNVs) or single-nucleotide polymorphisms (SNPs) associated with changes in a microbial abundance, researchers can identify the genetic factors shaping the human microbiome, which is increasingly recognized as an important contributor to human health and disease leading to the increased development of personalized medicine. Understanding the genetic basis of host–microbe interactions can additionally provide insights into the evolutionary history of such relationships that may have co-evolved for millions of years (Qin *et al.* 2022).

When performing mbQTL analysis there are a number of considerations not limited to multiple testing correction, normalization, and sparsity (Weissbrod *et al.* 2018). To date, there is an inconsistency in how mGWAS studies are analyzed due to differences in analytical methods, and a lack of reproducibility and standardization in the statistical framework (Weissbrod *et al.* 2018). Here, we present mbQTL, a Bioconductor package enabling reproducibility and standardization in this growing field for the analysis of microbial, viral, and eukaryotic quantitative trait loci.

## 2 mbQTL methods

Implemented are three approaches for microbe–host interaction testing. Our first approach (A) is based on the hypothesis that a particular SNV is associated with increases or decreases of bacterial abundance. Assuming abundance of every taxa is similar to gene expression quantitatively, the abundance of a taxa can be associated with the presence of a SNV. As an example, Mehta *et al.* (2020) show that *G. vaginalis*’ relative abundance in women is associated with the presence or absence of rs1229660. Our second hypothesis (approach B) is that there exists co-abundant bacterial operational taxonomic units (OTUs) associated with particular genotypes. As an example, Qin *et al.* (2022) identifies presence of a cluster of SNVs in a particular locus of human genome associated with *Bifidobacterium* and *Acinetobacterioum* genera. Finally, our third approach (C) assumes particular SNVs are associated with the presence or absence of specific taxa. Bartha *et al.* (2013) use this logic to identify associations between the microbial presence and SNV presence in both host and HIV genomes.

The first approach (A) employs multiple linear regression to examine the relationship between bacterial abundance and host SNVs. The user can design their own model matrix for each microbe–SNV pair to control for phenotypes of interest (Shabalin 2012).

The second approach (B) is a correlation-based method, scaling the correlation (ρ) between two taxa *x*, *y*, by the goodness of fit estimates ($R^{2}$). This approach will highlight bacteria associated with a polymorphic trait and find clusters of bacteria positively and negatively correlated with each other (Qin *et al.* 2022). Subsequently, we identify clusters of taxa that are associated with various SNVs across the dataset by evaluating and controlling for both the ρ and $R^{2}$ measures:

(1) $$\;{\hat{\rho }}_{x,y}=\frac{E[(x-\mu_{x})(y-\mu_{y})]}{\sigma_{x}\sigma_{y}}\times \frac{1}{R_{x}^{2}R_{y}^{2}}\;.$$

**Figure 1.**
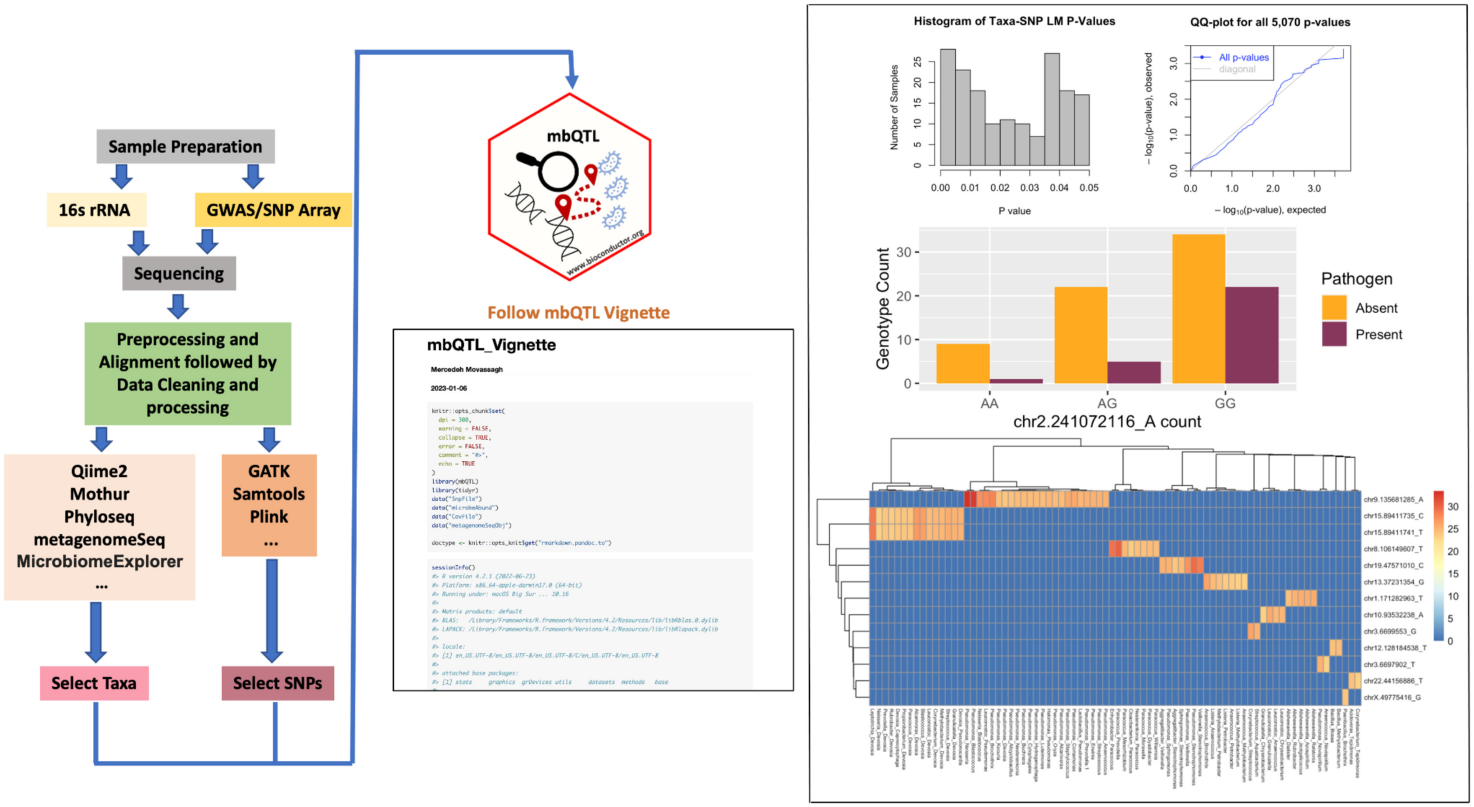
Overall workflow of mbQTL. Samples are prepared, sequenced, and preprocessed followed by filtering select taxa and SNPs are input. Further details are described in the package vignette. mbQTL provides convenient functions for plotting and identifying microbe-SNV/SNP relationships.

The correlation-based approach (approach B) allows us to demonstrate the relationships between SNVs and various microbes, as well as identify SNVs that are in linkage disequilibrium with each other and their expression patterns affect similar microbes as seen in Fig. 1.

In our final approach (approach C), we assume a binary relationship between each microbe and SNV pair and use a logistic regression model to determine the significant microbe–SNV relationships (Bartha *et al.* 2013).

## 3 Analysis workflow, results, and reproducibility reporting

User input includes three objects: (i) microbial abundance in the form of a MRexperiment object, (ii) a SNV matrix, which should be in the heterozygous format (0 = reference allele, 1 = heterozygous allele, 2 = homozygous alternate allele), and lastly (iii) a phenotype matrix to include covariates for multiple regression adjustment. For logistic regression, abundance thresholds are required.

Main functions for linear and logistic regression analysis are linearTaxaSnp(), histPvalueLm(), qqPlotLm() and logRegSnpsTaxa(), logitPlotSnpTaxa(). For the correlation based analysis the following functions are implemented, coringTaxa(), allToAllProduct(), taxaSnpCor(), and mbQtlCorHeatmap() for easy visualization of SNVs in linkage disqeuilibrium, e.g. chr15 in the package example dataset.mbQTL users can evaluate the performance of their analysis in approach A and C using the qqplot function, which compares expected versus observed *P*-values for the linear model, in addition to FDR and Bonferroni P reporting. In approach B, as the method reports on the strength of the SNV-taxa and taxa-taxa relationships, we provide functions for flexible cutoff selection for the highest probability correlation estimates. This enables users to evaluate the method’s performance using various cutoffs, allowing to correct for the occurrence of false positives. The package is on the Bioconductor platform to allow extended use and maximum reproducibility of results from various labs that use this package in the research community.

Finally, the mbQTL package allows for the explanation of linear, correlative and binary investigations of microbe–SNVs estimation.

## 4 Conclusion

We present mbQTL, an R package designed to provide a convenient method for microbe-SNV association analysis and visualization. Our approach incorporates three different models to identify significant microbe–SNV relationships: linear regression, correlation estimation ($\hat{\rho }$) and logistic regression. Originally developed for microbe–SNV association analysis, the statistical methods used in mbQTL can also be applied to the identification of other pathogens such as viruses, parasites, and fungi through RNA sequencing. This demonstrates the versatility of mbQTL and its potential to be utilized in conjunction with other NGS methods for pathogen identification and SNV association analysis.

## Data Availability

All simulated data is publicly available on https://zenodo.org/record/8250779.
